# Rare Diaphragmatic Hernias in Adults—Experience of a Tertiary Center in Esophageal Surgery and Narrative Review of the Literature

**DOI:** 10.3390/diagnostics14010085

**Published:** 2023-12-29

**Authors:** Dragos Predescu, Florin Achim, Bogdan Socea, Mihail Constantin Ceaușu, Adrian Constantin

**Affiliations:** 1 Faculty of Medicine, Carol Davila University of Medicine and Pharmacy, 050474 Bucharest, Romania; drpredescu@yahoo.com (D.P.); bogdan.socea@umfcd.ro (B.S.); mihail.ceausu@umfcd.ro (M.C.C.); dradiconstantin@yahoo.com (A.C.); 2 General and Esophageal Clinic, “Sf. Maria” Clinical Hospital, 011192 Bucharest, Romania; 3 Department of Surgery, “Sf. Pantelimon” Clinical Emergency Hospital, 021659 Bucharest, Romania; 4 Department of Histopathology, Alexandru Trestioreanu” National Institute of Oncology, 022328 Bucharest, Romania

**Keywords:** non-hiatal diaphragmatic hernias, Bochdalek hernia, Larey-Morgagni hernia

## Abstract

A rare entity of non-hiatal type transdiaphragmatic hernias, which must be clearly differentiated from paraoesophageal hernias, are the phrenic defects that bear the generic name of congenital hernias—Bochdalek hernia and Larey-Morgagni hernia, respectively. The etiological substrate is relatively simple: the presence of preformed anatomical openings, which either do or do not enable transit from the thoracic region to the abdominal region or, most often, vice versa, from the abdomen to the thorax, of various visceral elements (spleen, liver, stomach, colon, pancreas, etc.). Apart from the congenital origin, a somewhat rarer group is described, representing about 1–7% of the total: an acquired variant of the traumatic type, frequently through a contusive type mechanism, which produces diaphragmatic strains/ruptures. Apparently, the symptomatology is heterogeneous, being dependent on the location of the hernia, the dimensions of the defect, which abdominal viscera is involved through the hernial opening, its degree of migration, and whether there are volvulation/ischemia/obstruction phenomena. Often, its clinical appearance is modest, mainly incidental discoveries, the majority being digestive manifestations. Severe digestive complications such as strangulation, volvus, and perforation are rare and are accompanied by severe shock, suddenly appearing after several non-specific digestive prodromes. Diagnosis combines imaging evaluations (plain radiology, contrast, CT) with endoscopic ones. Surgical treatment is recommended regardless of the side on which the diaphragmatic defect is located or the secondary symptoms due to potential complications. The approach options are thoracic, abdominal or combined thoracoabdominal approach, and classic or minimally invasive. Most often, selection of the type of approach should be made taking into account two elements: the size of the defect, assessed by CT, and the presence of major complications. Any hiatal defect that is larger than 5 cm^2^ (the hiatal hernia surface (HSA)) has a formal recommendation of mesh reinforcement. The recurrence rate is not negligible, and statistical data show that the period of the first postoperative year is prime for recurrence, being directly proportional to the size of the defect. As a result, in patients who were required to use mesh, the recurrence rate is somewhere between 27 and 41% (!), while for cases with primary suture, i.e., with a modest diaphragmatic defect, this is approx. 4%.

## 1. Introduction

A small number of cases in adults present particular variants of diaphragmatic hernia of the non-hiatal type. The generic name of congenital hernias, otherwise the most common alternative to diaphragmatic hernias, can be explained by the appearance of these pathologies at pediatric ages, with the substrate being the relatively simple etiology: the presence of preformed anatomical openings, which either do or do not allow passing from the thorax to the abdomen, or the reverse, of anatomical elements (e.g., internal mammary vessels). Also, in the same class of non-hiatal diaphragmatic hernias but outside of the congenital origin, a somewhat rarer group is described, representing about 1–7% of their total: the acquired traumatic variant, frequently of a contusive-type mechanism, which produces diaphragmatic strains/ruptures [1]. All these hernias must be clearly differentiated from paraoesophageal hernias, with the latter often being due to complications of previous fundoplications or surgical interventions in the area of the hiatal orifice.

One thing is certain: adult patients are usually rare cases that are discovered incidentally, without statistical consistency. The lack of a statistical basis, even in centers with experience in thoraco-abdominal border surgery, gives way to arbitrariness in the management of these patients.

We note from the beginning that this review concerns the diagnostic and therapeutic issue of adults with rare diaphragmatic hernias but which, due to their congenital etiology, will also have a series of aspects that will be present from the childhood period and connected with those. The pediatric component was not a goal of this review, but it has been consequently invoked and presented sequentially only to allow a better understanding of the etiological mechanisms and the clinical impact in adults, with possibilities of diagnosis and therapeutic conduct.

The purpose of the article concerns several factors. First of all, a series of unclear aspects, dilemmas, and controversies from both the specialized literature and clinical experience are targeted and analyzed.

Secondly, elements that had the confirmation of time in terms of surgical management, both diagnostic and therapeutic, are presented and reinforced.

Third, and perhaps the most important objective, are answers to some questions from clinicians: What are the clinical elements that can suggest a diaphragmatic hernia in an adult? What are the assessment and diagnosis possibilities and what are the limits and controversies of these diagnostic techniques? What are the current therapeutic options, what are the advantages and disadvantages of some of these methods, what risks and complications do they present, and how and why can we choose one therapy or another? It should now be mentioned that the authors are specialized in upper digestive surgery.

## 2. Materials and Methods

In order to prepare the scientific material, this article is based on an analysis of data considered relevant from two directions. The first source was based on the experience accumulated from cases admitted, diagnosed, and treated in the General and Esophageal Surgery Clinic, UMF Carol Davila, Bucharest, in the period 2000–2022, without performing a detailed analysis of each case. A second source of scientific material was based on an extensive search in the international databases Embase (Excerpta Medica Database), PubMed Central (PMC), Cochrane Library, and MEDLINE Complete (EBSCO) starting from the year 2000, using some key words: rare diaphragmatic hernias, diaphragmatic hernias, non-hiatal diaphragmatic hernias. As a more sensitive search strategy, we used additional keywords: congenital diaphragmatic hernias, Bochdalek hernia, Larrey-Morgagni hernia, post-traumatic diaphragmatic hernias.

Regarding the data from the literature, based on the keywords, only these articles were identified and considered eligible, with the vast majority of them being clinical case reports—201, with the remaining 36 reported as original articles and 13 reviews. Data regarding diagnostic and therapeutic management in newborn and pediatric patients (age < 16 years) were excluded from this material. Two authors (AC and DP) selected the articles considered relevant, preferring peer-reviewed articles from highly ranked journals written in English. The decision to select an item was made by agreement of the two. The reference list from each selected article was screened for additional relevant information. We excluded unpublished data from abstracts contained in volumes from various congresses or conferences, and we excluded papers that were not in English. The two authors focused on data regarding sex, age, anamnestic diagnosis, symptomatology, paraclinical diagnosis, location and dimensions of the diaphragmatic defect, surgical indication—emergency or chronic, type of surgical approach—classic or minimally invasive, abdominal thoracic or mixed, sac resection, type of repair, use of mesh and type of mesh, postoperative complications, postoperative mortality, long-term outcomes (especially recurrence).

## 3. Results

Between 2000 and 2022, 18 cases of non-hiatal diaphragmatic hernias were identified in the clinic’s electronic archive. The extreme ages were 26 years (minimum) and 78 (maximum). Anamnestically, six post-traumatic hernias were identified in the oldest patients in the analyzed group, with the time interval from the traumatic event to presentation in the clinic being on average 40 months. Of the congenital hernias, five were postero-lateral Bochdalek hernias, three Morgagni hernias and four Larrey hernias. The majority of the surgical techniques were performed only via the abdominal approach, twelve via the open approach, and six laparoscopically. In three cases, a simple suture of the defect was performed (one Bochdalek hernia, one Larey hernia, and one Morgagni hernia) and in the remaining fifteen, alloplastic material was used—thirteen with dual mesh and two with biological mesh. In two cases, the mesh had a substitution role due to the large size of the defect, over 12 cm in diameter.

We had no indications for emergency operations, with all operations being elective. Long-term follow up was possible only in 15 patients at 6 months, respectively, and 14 patients one year postoperatively. After one year, we identified two recurrences after laparoscopic interventions without mesh placement, one Bochdalek hernia and one Morgagni hernia, both with a 7–8 cm^2^ defect. One hernia was repaired minimally invasively with mesh. The second case, with many co-morbidities, refused surgical treatment.

### 3.1. Non-Hiatal Congenital Diaphragmatic Hernia in Adults

The congenital origin is documented by the embryological development of the diaphragm. Although the data are relatively insufficiently known, diaphragmatic formation seems to begin between weeks 8 and 12, with four elements competing for its development: the septum transversum (mesodermal origin), the pleuro-peritoneal membranes, the mesoesophagus, and the muscles of the abdominal wall [2]. The final three muscular components of the diaphragmatic structure, from posterior to anterior, are the pars lumbaris (the most consistent), the pars costalis, and the pars sternalis. The three muscular elements converge towards a central area, known as the central tendon. The areas of intersection between the three muscle groups enable the appearance of weak areas (gaps), covered only by the peritoneum and pleura, and two fascial elements—fascia transversalis and frenopleural—which are a place of choice for the appearance of transdiaphragmatic hernias. The two main weak areas are described at the posterior level of the diaphragm, through incomplete fusion between the pars lumbaris and pars costalis (Bochdalek hernia), respectively, on the anterior, parasternal, right (Morgagni), or left (Larey) diaphragm, through a closing defect between the pars costalis and pars sternalis. These anterior Larey-Morgagni defects, corresponding to a triangular area, the sternocostal triangle, are bounded by the sternum, diaphragm, and pericardium and are traversed by the internal mammary pedicles (vessels and lymphatics). Congenital defects of diaphragmatic scoliosis have different degrees, with incomplete sealing of the communication between the thoraco-abdominal compartments. The presence of an important defect results in early visceral migration from the abdominal area, with a positive pressure, to the thoracic area, with an alternately negative pressure value. Major birth defects usually occur in the intrauterine period, being reported 1 in 2100–5000/births. The main impact is pulmonary, compromising its normal development [3]. The presence of a polyhydramnios is seen in about 80% of cases, being an unfavorable prognostic factor, with only 11% of these fetuses surviving [4].

Small defects from the childhood period can evolve dimensionally under the aggression, especially with a combination of some factors that abnormally increase intra-abdominal pressure (traumas, chronic coughs, severe constipation, pregnancy, etc.), explaining the appearance of these congenital hernias in adults as well. Another mechanism is the tissue degradation that occurs with age, with the laxity of the musculo-aponeurotic elements favoring the progressive weakening of the diaphragmatic content on an already precarious congenital background. A rare form, also congenital, is diaphragmatic eventration. This specifically has an area of diaphragmatic muscle aplasia, usually on the dome, through which the abdominal viscera are engaged in a hernia sac formed by the pleura, peritoneum, and possibly diaphragmatic tendinous elements. The simultaneous presence of cardiac and pulmonary developmental abnormalities (tracheomalacia, hypoplasia), various visceral occlusion defects (ectopies, malrotations), and congenital syndromes such as Beckwith–Wiedemann, Poland, etc., are reported in [5].

The etiology remains unknown but is probably multifactorial. Congenital diaphragmatic hernias appear in 50–70% of cases as unique defects. The rest of the cases are associated with other morphological anomalies and/or genetic changes. The morphological changes can be cardiovascular, neurological, or musculoskeletal.

Cardiovascular malformations (ventricular septal defects, atrial septal defects, tetralogy of Fallot) occur in 11–15% of isolated cases of congenital diaphragmatic hernias (25–40% of all cases of congenital diaphragmatic hernias). Central nervous system abnormalities such as neural tube defects and hydrocephalus occur in 5–10% of cases. Limb abnormalities such as polydactyly and syndactyly occur in approximately 10% of cases [6].

Chromosomal abnormalities, including aneuploidies, chromosomal deletions/duplications, and complex chromosomal changes, are identified in 10–35% of cases of prenatally diagnosed diaphragmatic hernias with multiple morphologic changes. Trisomy 13, 18, 21, and 45, X are the most commonly associated aneuploidies [7]. In addition to karyotype abnormalities, in 3.5–13% of cases with congenital diaphragmatic hernias without other morphological changes, microdeletions or microduplications are identified. Pallister–Killian syndrome or tetrasomy 12p caused by isochromosome 12p is one of the most common karyotype changes. An underlying genetic syndrome is present in approximately 10% of cases of congenital diaphragmatic hernia and Fryns syndrome is the most commonly diagnosed syndrome [7,8].

There is some documentation on the role of environmental factors in the occurrence of congenital diaphragmatic hernias. The plasma level of retinol and retinol-binding protein seems to be directly related to the occurrence of diaphragmatic defects [6].

### 3.2. Bochdalek Congenital Hernia

Described for the first time by Bochdalek in 1848, this type of hernia is responsible for about 90% of congenital hernias and is found in cases at rate between 0.17 and 6% in large population studies [2,9,10]. A controversy of this hernia concerns the preferred seat, left or right, in the adult patient. Although a predominantly left-sided involvement is accepted, a number of newer studies [9] contradict these views, proving a predominantly right-sided involvement. The dorsal position explains the engagement through the hernial orifice, especially of elements from the posterior abdomen: retroperitoneal fat, kidneys, or, exceptionally, other abdominal viscera (liver, spleen, small intestine, colon) [11,12]. The statistics from the literature, quite modest in terms of the number of cases accumulated in a surgical service, most of the time being case reports, show that the most common herniated organs on the left side are the stomach and the great omentum, but other situations are also described in which the small intestine, colon, spleen, and left liver are identified. On the right side, the kidney and pancreas are the most involved [13].

Apparently, the symptomatology is heterogeneous, depending on the location of the hernia, the dimensions of the defect, which abdominal viscera is involved through the hernial opening, its degree of migration, and if there are phenomena of volvulation/ischemia/obstruction.

Committed right-sided hernias appear more clinically silent, while left-sided hernias are more likely to present overt disease. According to some authors, the cause is most likely to be found in the dimensional variations in the hiatal defect, with the right one being statistically identified to have larger dimensions than the one on the left. The question naturally arises as to why larger sizes come with paucisymptomatic manifestations. On the one hand, the larger dimensions facilitate the visceral upside-down game, and on the other hand, the tourniquet effect of the hernial orifice is modest, with little impact on the herniated organs [14].

The presence of a large hernia increases the risk of respiratory failure. In the adult population, respiratory failure can be a consequence of atelectasis and pulmonary compression produced by a voluminous hernia, most often in hernias through the left hemithorax [15].

Not infrequently, especially in adults, the symptomatology has a digestive character, with acute or chronic phenomena [11] that are most often related to the harassment brought by herniation to the physiological transit, possibly combined with phenomena of visceral ischemia. The association of a volvulus can lead to somewhat more present symptoms, with regurgitation, reflux, and significant postprandial discomfort. Severe digestive complications such as strangulation, volvus, and perforation are rare and are accompanied by severe shock, suddenly installed after several non-specific digestive prodromes. Also interesting is the presence of a significant number of cases without symptoms; the performance of an imaging investigation (thoracic pulmonary radiology, contrast radiology, tomography) routinely or for other clinical manifestations may reveal the presence of a hernia. An example is traumatic injury of the contusive type, which can precipitate the diagnosis by setting up a specific symptomatology, a consequence of the local debilitating effect, with an increase in the size of the defect.

The method of diagnosis is imaging, but clinical presentation also plays an important role. Auscultation may reveal attenuation of the vesical murmur due to identification of hydro-air sounds. Fingerhut describes three possible clinical manifestations: (1) the appearance of respiratory symptoms, especially postprandial, (2) worsening of abdominal and/or thoracic symptoms when supine, and (3) physical exertion precipitates the onset of abdominal symptoms [16].

Diagnostic imaging recognizes two different scenarios of medical indication: (01) recommendation for radiological investigations in patients presenting with respiratory and/or digestive symptoms (reflux, digestive discomfort, etc.) and (02) incidental discovery in asymptomatic patients. In exceptional situations, medical emergency is the major complication of diaphragmatic hernias (strangulation ± volvulation, perforation).

**Figure 1:**
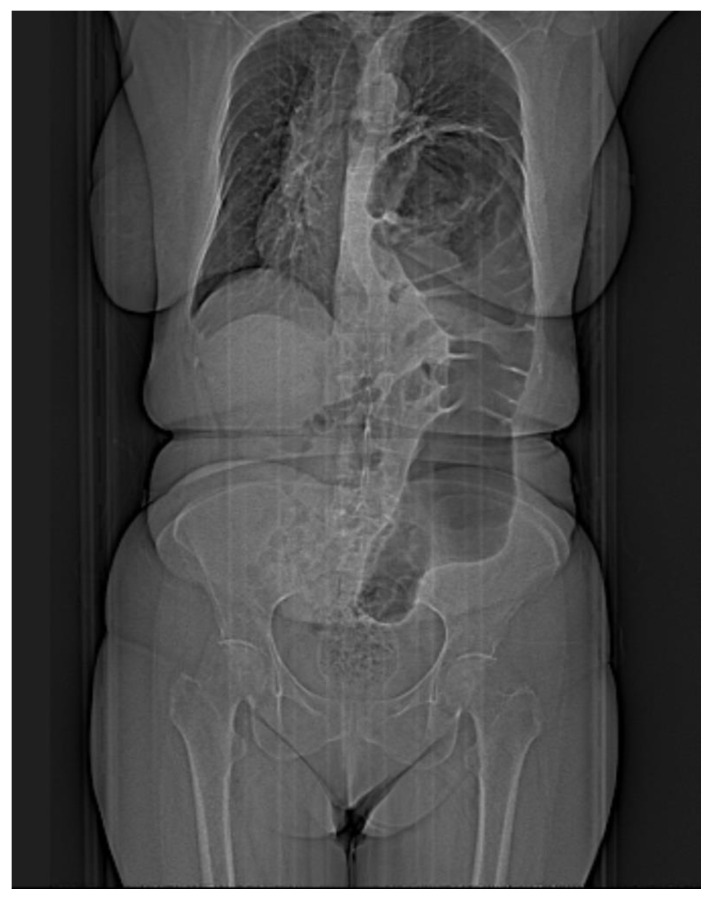
**Simple imaging assessment—radiography.** The presence of the colon, ascended transdiaphragmatically through a Bochdalek hernia, at the level of the left hemithorax is noted.

Chest X-ray is most often the first radiological investigation performed in these patients (90%). Chest and abdominal X-ray is recommended to be performed in multiple incidences, including the Trendelenburg position. Specific radiological signs are opacification of the lung bases, especially of the posterior fields depending on the contents of the hernial sac (Figure 1), intrathoracic localization of the abdominal viscera, and left hemidiaphragm elevation (more than 4 cm) with or without atelectasis. In general, diagnosis is more difficult when the hernial orifice is located on the right hemidiaphragm.

**Figure 2:**
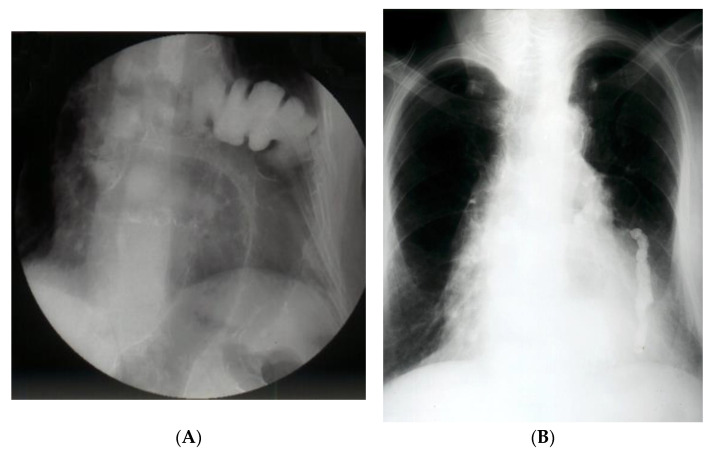
(**A**). **Contrast radiology—irrigography.** The intrathoracic presence of the colon is noted through a Bochdalek hernia (**A**) or post-traumatic (anamnesis) hernia (**B**).

The contrasting exam has its advantages in allowing the identification of the digestive viscera by directly opacifying the herniated organ (Figure 2A,B). Furthermore, it can identify upside-down movements, highlighting fixation, incarceration, or even visceral strangulation.

**Figure 3:**
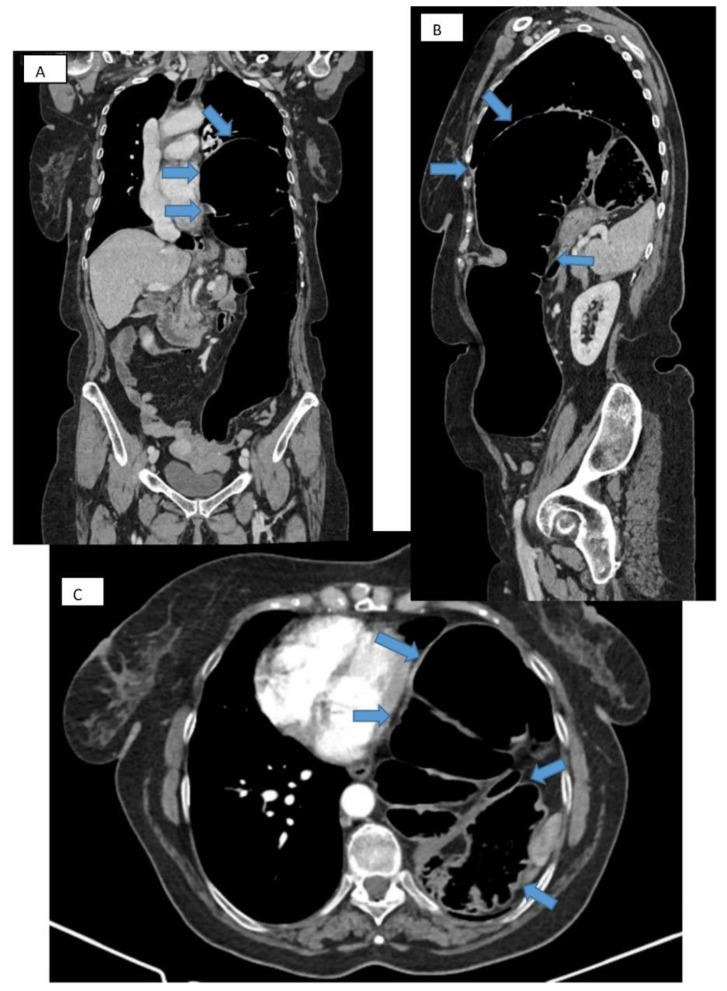
**CT thorax with contrast.** Frontal (**A**), sagittal (**B**), and transverse (**C**) section showing large diaphragmatic defect (Bochdalek hernia) with intrathoracic herniation of the colon.

Computed tomography refines the diagnosis by accurately identifying the location, dimensions of the defect, the hernial sac, and the intrathoracic herniation of viscera [17] (Figure 3A–C). Tomographic imaging represents a key element in establishing therapeutic decisions. CT has a sensitivity for left-sided injuries that is greater (78–100%) than for right-sided injuries (50–79%). The presence of a pleural effusion, a pulmonary atelectasis, simultaneously with the identification of digestive viscera at the thoracic level is revealing for diagnosis.

In addition, the special quality of CT allows a quality differential diagnosis, with a series of diaphragmatic or mediastinal and neurogenic tumors, etc., being excluded as Bochdalek hernias. A well-defined diaphragm without a loss in contour, continuously, leads to a different diagnosis (e.g., a fatty mass in this case is more likely a lipoma than a hernia).

In a group of 13,138 patients with known malignancy who underwent abdominal CT for oncological monitoring, the incidence of Bochdalek hernia in asymptomatic adults was 0.17%. In 68% of patients, the diaphragmatic defect was on the right side. In total, 77% were women [18].

Surgical treatment is recommended regardless of the side on which the diaphragmatic defect is located or the secondary symptoms due to potential complications [19]. The question naturally arises whether for asymptomatic patients, discovered incidentally, this recommendation is maintained. Almost all of the articles and studies in the literature recommend surgical intervention and only in exceptional cases, such as the presence of significant comorbidities, recommended abstinence [20,21].

Despite the fact that surgery is the main treatment, anatomical changes can vary from case to case and therefore the surgical treatment must be individualized based on the imaging findings.

**Figure 4:**
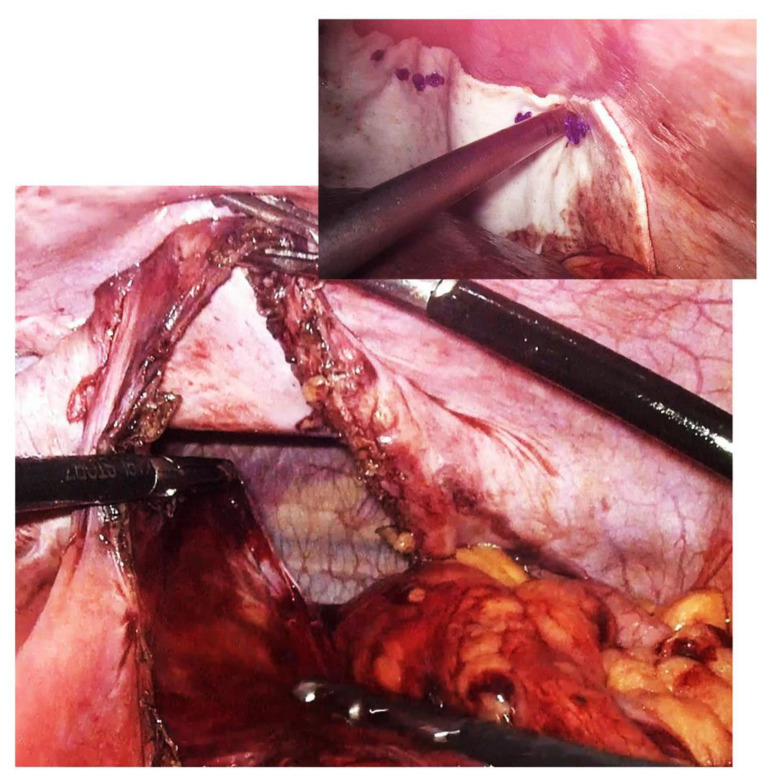
Intraoperative aspect of laparoscopic surgery for Bochdalek diaphragmatic hernia—the diaphragmatic defect of about 18 cm is highlighted. In the smaller image is the final appearance after primary suture of the defect and reinforcement with biological mesh.

Approach options are the thoracic, abdominal, or combined thoraco-abdominal approach, classic or minimally invasive [22]. The advantages of the thoracic approach over the abdominal approach are direct visualization of the hernial contents and non-negative intrapleural pressure, which facilitates reduction. Through the thoracic approach, adhesions between herniated viscera and the lung or pleura can be safely dissected, although few intrapleural adhesions have been found in previous reports. On the other hand, the thoracic approach in its classic version has the disadvantage of a much more “aggressive” technique for the patient; it forces selective intubation, a surgical gesture limited by the intercostal access, an often much more complex resuscitation, and a difficult, long recovery. In contrast, the advantage of the abdominal approach is the easier recognition and management of possible strangulated bowel loops or concomitant abnormalities of the abdominal viscera [23]. The abdominal approach is more commonly used than the thoracic approach and may be more appropriate in complicated cases with strangulation, ischemia, or visceral abnormalities. Minimally invasive thoracoscopic, laparoscopic, or robotic surgical interventions have slowly become the procedures of choice and are increasingly used on both sides of the diaphragm [24] due to their well-known advantages (Figure 4). The presence of strangulation, volvulation, and perforation are formal contraindications to a minimally invasive technique, but here it must be judged on a case-by-case basis [25,26,27].

Relatively recently, a report from an extended work group—Congenital Diaphragmatic Hernia Study Group (CDHSG)—was published in which the statistical base included 3067 patients operated on between 2007 and 2015, with the vast majority through open surgery (84%) versus minimally invasive surgery (16%) [28].

Placing the patient on the operating table in laparoscopic surgery can also be in lateral decubitus in the anti-Trendelenburg position [29].

The principles of the surgical technique do not differ essentially from other abdominal containment defects: reduction in the hernia content and restoration of the anatomy, resection of the hernia sac, and closure of the hiatal defect and possibly reinforcement or even substitution with an alloplastic material (mesh).

Reducing the content can be relatively easy, but the presence of intra- and extrasaccular adhesions complicates the procedure. In the thoracic approach, reduction is characterized by a visible “push-back” technique, while in an abdominal approach, the method is a “pull-back”. Cases are reported in which, through the thoracoscopic approach, the simple insufflation of gas reduced the contents back into the abdomen [30,31].

A degree of incarceration or even strangulation makes dissection, mobilization, and release of the contents difficult, forcing the surgeon to make risky moves or various technical artifices.

There is much debate in the literature regarding whether or not to resect the hernial sac. The vast majority recommend resection for two reasons: increased risk of recurrence and its transformation into a seroma. We believe that the surgical attitude must be nuanced, i.e., if dissection is possible and does not appear to be very laborious, does not extend excessively, and if the risks of visceral injuries are reduced, then resection, at least partial, is advisable. Whenever the risks outweigh the benefits, it should be abandoned. Some authors even note the disappearance of the bag quickly post-operatively, without any inconvenience [32].

**Figure 5:**
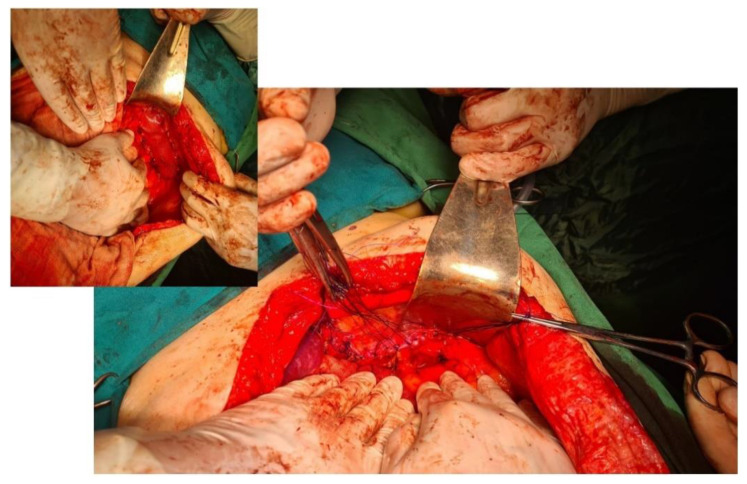
**Intraoperative aspect.** Primary suture of the diaphragmatic defect for a Bochdalek hernia. In the smaller image is the final appearance after reinforcing the suture of the defect with dual mesh.

The diaphragmatic defect is usually closed by simple suture, with nonabsorbable sutures for minor defects [33,34]. Any hiatal defect that is larger than 5 cm^2^ (the hiatal hernia area (HSA)) has a formal recommendation for reinforcement—if the edges of the defect can be sutured—with either mesh or replacement if aggrandizement is not possible (Figure 5). Beyond the large size of the hernia, the use of mesh can also be recommended for cases with a diaphragm with a fragile texture, such as in patients with extreme obesity [35,36].

Currently, there are no data to suggest the superiority of a certain type of mesh, whether that is synthetic or biological. The latest data show an apparent superiority of biological meshes on the one hand due to excellent visceral tolerance and on the other due to a low rate of recurrence over time [37].

The recurrence rate is not negligible. Statistical data show that the period of the first postoperative year is prime for recurrence, with the rate decreasing progressively with the passage of time. Proportionally, the larger the size of the defect, the greater the risk of recurrence. Consequently, in patients who were required to use the mesh, the recurrence rate is somewhere between 27 and 41%, while for cases with primary suture, that is, with a modest diaphragmatic defect, this is about 4%. The type of approach has an impact on recurrence: between 0 and 13% in open surgery and, despite the obvious advantages, approximately 6–39% for minimally invasive surgery [38,39,40].

### 3.3. Larrey-Morgagni Congenital Hernia

The anatomist Giovanni Morgagni first described this type of hernia in 1769. It occurs extremely rarely in adults, with an incidence of 2–4% of all congenital diaphragmatic hernias. It is more frequent in women with a ratio of 2:1. Its anterior placement, retroxiphoid, corresponds to a weak triangular area over which it etiologically overlaps and an incomplete migration of muscle fibers to close the parietal defect. A series of etiologically involved predisposing factors are similar to those of other abdominal hernias and result in increased intra-abdominal pressure (obesity, pregnancy, trauma, chronic coughers, etc.) [41]. Right involvement is the most frequent (over 90%), which is probably a consequence of superior reinforcement on the left side due to pericardial attachments. The occurrence, especially in women, is interesting and the presence of bilateral defects is an exception [42]. The variant, described in children, that associates a hiatal hernia with an intrathoracic stomach is also rare. The hernia content seems to depend on the age of diagnosis: in newborns, presence in the liver, stomach, and small intestine have been found, while in older children, they are found mainly in the small intestine. In adults, the presence of epiloon is often reported, exceptionally in the stomach, small intestine, or liver [3].

The symptomatology has a high similarity to other non-hiatal diaphragmatic hernias. Often, symptoms can be completely absent (10–30%) or have the appearance of retrosternal pain, with variable intensities and duration. Inconsistent pain of low intensity will not alarm the patient and will consequently delay the diagnosis. Associated with or as the only sign, ventilatory dysfunction has been described, especially in right or digestive hernias but also frequently in those on the left side. The most common symptoms are respiratory disorders (44.8%), abdominal pain (39.7%), and nausea and vomiting (30%). Acute phenomena such as content strangulation and, as a consequence, intestinal occlusion vary, featuring in between 7 and 20% of cases [43].

Diagnosis is suggested via imaging investigation (face, profile, oblique) through standard radiology with the identification of a fat mass to the right of the cardiophrenic angle, the presence of hydro-air levels, or a visceral mass of the digestive type. Contrast studies highlight gastro-enteric malposition, located supradiaphragmatically. Differential diagnosis to other types of diaphragmatic hernias or possible tumors (lipoma, teratoma, sarcoma, etc.) requires the association of CT or MRI, only for variants that confirm the character of a Morgagni hernia [44,45].

The only established treatment for Morgagni hernias in adulthood is surgery for both symptomatic and asymptomatic patients. Surgical repair of the hernia is required in all patients because of the potential risk of strangulation/occlusion, which, although low, is reputed to be serious. Although many surgical approaches have been described, there is no consensus on a standard approach, mainly because of the rarity of this entity [43].

The laparoscopic/thoracoscopic approach is preferred in elective cases when relevant expertise is available. The option for a laparoscopic technique is argued, for strictly technical reasons, by the fact that most often the hiatal defect is not an important one and the visceral adhesions are modest and so, consequently, a reduction in the content is not a difficult maneuver. Consequently, laparoscopy is not a demanding technique. Excision of the sac comes with its controversies, with more than half of surgeons giving up on its excision. There is an argument relating to a prolonged operating time, possible risks of the excisional gesture, lack of an increase in the recurrence rate, or a minimal risk of cystic transformation. With or without excision of the sac, the defect is sutured and pre-peritoneally reinforced with alloplastic material by using sandwich mesh or dual or hybrid mesh placed directly over the defect [46,47].

We believe that the use of mesh must be conditioned by the size of the defect, the quality of the tissues for closing the hernial defect, and the tension of the suture. A small defect, most often Morgagni hernias, with tendinous, fibrous edges that react relatively easily to suture does not necessitate the placement of mesh. The persistence of some causes that have an etiological role—chronic coughers, obesity, chronic constipation, etc.—tilts the balance in favor of placing a mesh. Doubts relating to the use of prosthetic materials give way to a variant of reinforcement with falciform ligament, with very good results reported by the authors of [48,49,50].

Intraoperatively, endoscopic assistance can be of real benefit to the surgeon during dissection and anatomy restoration.

The management of emergency cases or of any difficulties during the laparoscopic time justifies an open approach, also allowing the resolution of complications from the digestive tube, for example, gangrene by strangulation; in any case, both approaches do not differ significantly in morbidity and mortality rate. Transthoracic techniques have indications and contraindications similar to those of the Bochdalek hernia, having indications especially for recurrent hernias or the presence of intrathoracic lesions/pathologies [51,52].

### 3.4. Post-Traumatic Diaphragmatic Hernia (Acquired)

Post-traumatic diaphragmatic hernia was described for the first time by Sennertus as early as 1541 [53]. Three decades later, Pare documents a case of postmortem diaphragmatic hernia at the necropsy of a patient with a recent history of abdominal trauma [54]. The first surgical treatment of this pathological entity was reported by Riolfi in 1886 and Naumman in 1888. Diaphragmatic injuries can represent up to 5% of the causes of hospitalization in trauma services [55]. However, the incidence of post-traumatic diaphragmatic hernias varies depending on the reports but is between 0.8 and 5%, with less than 2.7% of cases diagnosed in the first 4 months after the trauma. Spontaneous healing of a diaphragmatic rupture has not been reported [56].

Most of the time, the mechanism is a direct one of a contusive type, with a sudden increase in intra-abdominal pressure which mainly affects the left hemidiaphragm [57]. Its explanation comes from the protective role offered by the presence of the liver on the right side, which has a buffer effect. In corollary, it has been observed that the presence of a right hernia is accompanied by a much more severe traumatic lesion balance than that found in left hernias. Specific to blunt trauma is a usually large defect, larger than 10 cm, located especially in areas with predisposition (junction of muscle groups with the central tendon) and the presence of other visceral injuries (liver, spleen, kidneys) is also possible. Much rarer are penetrating injuries (stab wounds, gunshot wounds) that are typically 1–2 cm in size or iatrogenic (ex labor). Disengagement of the diaphragm from the chest wall is another variant encountered [58].

Diagnosis is relatively difficult due to the patient’s lack of awareness or cooperation, either by masking the symptomatology or by other more severe or important signs. Standard radiology can detect elevations of the diaphragm, its stretched appearance, and opacification of the lung fields, but the signs are difficult to interpret for many reasons (pre-existing or post-traumatic lung pathologies). CT enables diagnosis (rate between 66 and 80%) by visualizing the diaphragmatic defect and by the presence of intrathoracic abdominal viscera, immediately post-traumatic. Diagnostic doubts require MRI. Small tears/lacerations may remain inconspicuous but, over time, after months or even years, especially through the association of predisposing factors, they can lead to diaphragmatic hernias. In these cases, over time, symptoms develop with progressive intensity, with retrosternal pain, respiratory and possibly cardiac disorders, and transit disorders. The risk of occlusion by strangulation with necrosis ± perforation greatly worsens the prognosis [59].

This type of diaphragmatic hernia is usually described more frequently in men and in only 13% of cases is it on the right side. The stomach and colon are the most frequently herniated viscera due to their mobility and relationship with the diaphragm [60].

The surgical approach for chronic post-traumatic diaphragmatic hernias includes the transabdominal, transthoracic, and combined approach, with an increasing role of minimally invasive techniques. There is no consensus on the preferred approach. Classically, chronic large posttraumatic diaphragmatic hernias should be approached using thoracotomy to allow lysis of intrathoracic adhesions. A review of the literature on this subject establishes that the thoracic approach is 3 times more frequent than the abdominal approach (69% vs. 24%). Also, 10% of abdominal approaches required additional thoracotomy and 15% of thoracic approach cases required additional laparotomy. But, there was no statistical difference between either group [61].

The advantage of the abdominal approach compared to the thoracic one is due to both the optimal evaluation of the diaphragmatic injury and to the management of the incarcerated viscera.

Silva et al. highlights laparotomy as the most frequently used approach for emergency operated cases and thoracotomy for chronically operated cases [62].

The choice of approach ultimately depends on the associated lesions, as well as the experience and preference of the surgical team.

## 4. Discussions

Congenital and acquired diaphragmatic hernias in adults are, in each individual case, a particular medical situation. The data from the imaging and necropsy studies on large groups of patients do not show an incidence as low as we imagine and is between 0.41 and 6% (!) [2,9,10]. The question naturally arises as to why these cases are so rare in current medical practice. A first answer is given by the presence of symptoms. Whenever a symptom appears in childhood, the case is diagnosed and possibly has a therapeutic plan. But what happens when there are no symptoms? The standard evaluation of the child in their first years of life does not include investigating the existence of a diaphragmatic defect. The standard ultrasound evaluation can overlook a diaphragmatic defect in asymptomatic cases. If it is not diagnosed incidentally during a routine examination, the child will become an adult with a asymptomatic congenital diaphragmatic hernia. The presence of symptoms is always related to the size of the diaphragmatic defect, the hernia volume, and the type of herniated viscera. Small sizes have insignificant symptomatology or are even asymptomatic, explaining why these cases are most often discovered incidentally during routine explorations or for completely different pathologies. The paucisymptomatic character is the result of the lack of transhernia involvement of the abdominal viscera and/or a pleuro-pulmonary impact through dislocation. What is the size of diaphragmatic defects where visceral involvement occurs? That question has a difficult answer to define, with the equivocation being characteristic. In our experience, the smallest defect was approx. 4 cm^2^ (3 × 1.5 cm) and the largest was approx. 100 cm^2^ (18 × 6 cm). We have found in the literature some recommendations that establish a connection between the size of the defect and the surgical treatment options but not between the size of the defect and symptomatology [33,34,35,36].

Diagnosis of diaphragmatic hernias is challenging and is based on X-ray and imaging contrast studies. Dimensional assessment of the diaphragmatic defect is accurate only through CT assessment [24,25]. The location of the diaphragmatic defect, important anatomical details, the volume of the hernia, the mass effect created by the visceral mass and the cardio-pulmonary impact, ischemia phenomena, etc., are all aspects of information that could refine a complete diagnosis and a surgical decision. A complete diagnosis allows the most correct therapeutic conduct. We prefer an abdominal, classic, or minimally invasive approach.

**Figure 6:**
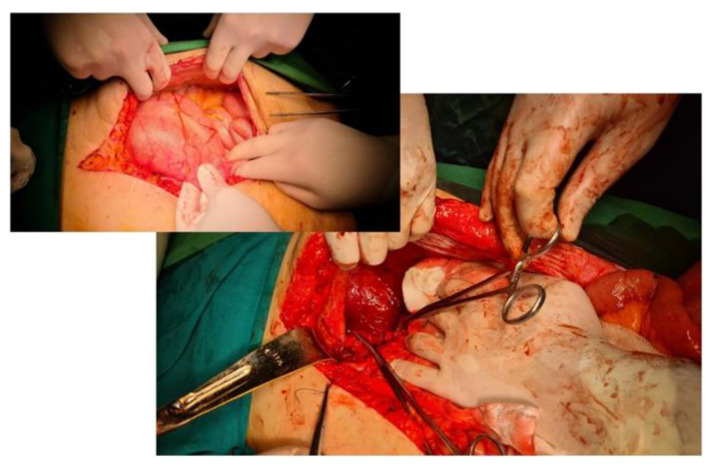
**Intraoperative aspect**. Large diaphragmatic defect of about 16 cm, Bochdalek hernia. In the smaller image, the colon is engaged transdiaphragmatically and dilated, with incomplete obstruction.

Most often, selection of the type of approach should be made taking into account two elements: the size of the defect, assessed by CT, and the presence of major complications. A defect larger than 8 cm makes the minimally invasive approach difficult because, in these cases, the installation of a mesh becomes mandatory and its dimensions are large and difficult to handle and anchor (Figure 6).

**Figure 7:**
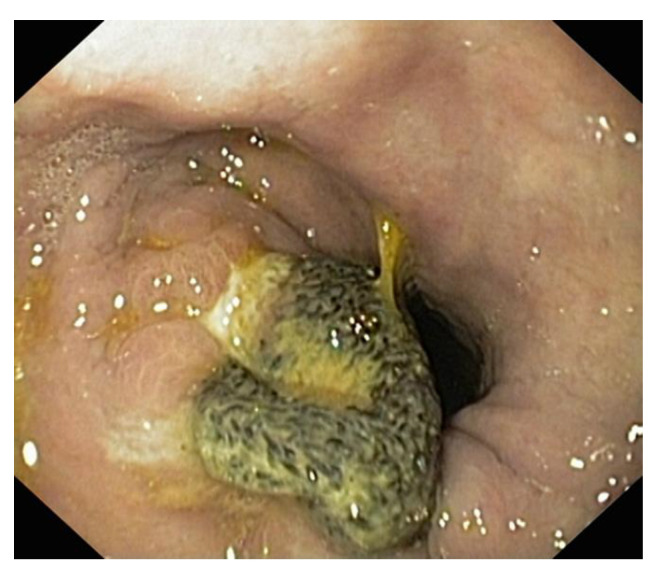
**Endoscopic aspect**. Maarlex-type mesh evacuated through the stomach after repair of a large Bochdalek-type diaphragmatic defect.

Trans-thoracic techniques require selective intubation, difficult intra- and postoperative resuscitation, are difficult access even in the thoracoscopic approach, and have higher rates of complications and prolonged recovery of the patient. Robotic surgery can be a solution, especially in small defects. If reduction in the content is mandatory, excision of the hernia sac raises discussions. We consider that its excision is mandatory, its preservation being validated only if it is used to cover the alloplastic material. We recommend the use of mesh, for consolidation or substitution, whenever the surface of the hernia hole is >5 cm^2^. We are of the same opinion as other authors [37,38] for dual mesh but especially for biological mesh, which is very well tolerated. Regular polypropylene mesh is not recommended as the risk of perforation is high (Figure 7).

## 5. Conclusions

Non-hiatal transdiaphragmatic hernias in adult, congenital or post-traumatic, are rare entities that must be clearly differentiated from paraoesophageal hernias and often constitute a diagnostic surprise, as noted in the specialized literature. A digestive symptomatology that associates respiratory and/or cardiac phenomena may raise suspicion of a congenital/post-traumatic hernia. Acute or severe symptomatology (volvulus, perforation, occlusion) can appear at any time, greatly complicating the therapeutic result. Diagnosis is often easy as imaging techniques can easily discover the presence of abdominal viscera at the thoracic level. Surgical techniques have evolved, often allowing a minimally invasive approach, with well-known advantages of this type of surgical management.

## Data Availability

Not applicable.
